# Protocol of the study for predicting empathy during VR sessions using sensor data and machine learning

**DOI:** 10.1371/journal.pone.0307385

**Published:** 2024-07-18

**Authors:** Emilija Kizhevska, Kristina Šparemblek, Mitja Luštrek

**Affiliations:** 1 Department of Intelligent Systems, Jožef Stefan Institute, Ljubljana, Slovenia; 2 Jožef Stefan International Postgraduate School (IPS), Ljubljana, Slovenia; University of Valencia: Universitat de Valencia, SPAIN

## Abstract

Virtual reality (VR) technology is often referred to as the ‘ultimate empathy machine’ due to its capability to immerse users in alternate perspectives and environments beyond their immediate physical reality. In this study, participants will be immersed in 3-dimensional 360° VR videos where actors express different emotions (sadness, happiness, anger, and anxiousness). The primary objective is to investigate the potential relationship between participants’ empathy levels and the changes in their physiological attributes. The empathy levels will be self-reported with questionnaires, and physiological attributes will be measured using different sensors. The main outcome of the study will be a machine learning model to predict a person’s empathy level based on their physiological responses while watching VR videos. Despite the existence of established methodologies and metrics in research and clinical domains, our aim is to contribute to addressing the gap of a universally accepted “gold standard” for assessing empathy. Additionally, we expect to deepen our understanding of the relationship between different emotions and psychological attributes, gender differences in empathy, and the impact of narrative context on empathic responses.

## Introduction

Empathy is a multifaceted and complex topic that has captured the interest of researchers across many different fields, including psychology, neuroscience, philosophy, sociology, and anthropology. Although there is no widely accepted definition of empathy, there is a consensus regarding its multidimensional nature. For instance, one of the most used theoretical models is the one that conceives empathy as encompassing a cognitive component (i.e., the capacity for understanding another person’s experience and perspective) and an emotional component (i.e., the ability to experience the emotional state of another person) [1, 2]. For the purpose of our research, we regard empathy as the ability or tendency to construct a working model of the emotional states of others and be sensitive to and experience other people’s feelings, while maintaining the awareness of the distinction between self and other [3].

Understanding the reasons why empathy is worth investigating can shed light on its significance in different domains. Here are some key points:

1. Importance of empathy in social relationships and cross-cultural differences in empathy: Empathy plays a crucial role in social interactions, enabling individuals to understand and experience the emotions of others. Moreover, cultural variations in empathy emphasize the diverse importance placed on empathy across different cultures. Research on empathy not only reveals its impact on forming and maintaining relationships, resolving conflicts, and fostering interpersonal connections but also provides insights into cross-cultural differences, helping us understand the factors that shape these variations and their implications for intercultural communication and understanding [4–6].
2. Prosocial behavior and altruism: Empathy has a strong link to prosocial behavior, such as helping, volunteering, and displaying altruistic acts. Investigating empathy can reveal how it influences individuals’ willingness to support others and contribute to the well-being of society [7, 8].
3. Mental health implications: Empathy deficits are observed in certain psychiatric disorders, including autism spectrum disorder, borderline personality disorder, and antisocial personality disorder. Research on empathy aids in understanding the underlying mechanisms of these deficits, potentially leading to improved diagnostic tools and therapeutic interventions [5].
4. Technological applications: As virtual reality and artificial intelligence continue to gain prominence, there is an increasing focus on developing technologies that have the ability to stimulate empathy. Empathy-based technologies are those that leverage the understanding of human empathy to enhance user experiences, communication, and interactions. They utilize techniques and features that evoke or facilitate empathic responses from users. For example, in the field of VR, empathy-based technologies may involve creating virtual scenarios that allow individuals to embody different perspectives, emotionally connect with characters, or experience situations that elicit empathy towards others [9].

Often, empathy and prosocial behavior are mistaken as the same concept. They are related, but differ in their nature and manifestation. As we mentioned before, empathy involves understanding and experiencing others’ emotions and experiences, fostering compassion and connection. Prosocial behavior, on the other hand, comprises actions aimed at benefiting others, such as kindness, cooperation, and altruism. While empathy can lead to prosocial behavior, they are distinct entities. Empathy serves as a cognitive and motivational precursor to prosocial actions, as it fosters a sense of responsibility and compassion, motivating individuals to help and support others [4, 7, 10–13].

While there is no universally agreed-upon “golden standard” for measuring empathy [14], several established methods and measures are commonly used in research and clinical settings, including self-report questionnaires, behavioral observation, psychophysiological measures and performance tasks.

1. Self-report questionnaires: Self-report questionnaires involve participants answering a series of questions about their thoughts, feelings, and behaviors related to empathy. These questionnaires typically assess self-perceived empathy levels and provide insights into individuals’ subjective experiences. However, one limitation is that self-report measures rely on participants’ self-perceptions and may be subject to response biases, such as social desirability or inaccurate self-assessment [15, 16].
2. Performance tasks: Performance tasks assess individuals’ empathic abilities through specific tasks or simulations designed to elicit empathic responses. These tasks often involve scenarios where participants must infer and understand others’ emotions or engage in perspective-taking. Performance tasks can provide more ecologically valid measures of empathy and overcome some limitations of self-report measures. Despite the need for careful design and the potential influence of factors such as task demands and individual cognitive abilities, the primary limitation of this method is its inability to measure the emotional dimension of empathy, potentially yielding lower levels of genuine empathic understanding. It is worth noting that individuals lacking empathy altogether may still possess effective advocacy skills [4, 17].
3. Behavioral observation: Behavioral observation involves directly observing and recording individuals’ empathic behaviors in real-world or controlled settings, without specific tasks designed to elicit empathy. Researchers use standardized coding schemes to quantify specific empathic behaviors, such as facial expressions, vocal tone, or prosocial actions. While behavioral observation provides objective data on actual empathic behaviors, it may be time-consuming, and the presence of observers might influence participants’ behavior, leading to potential reactivity biases [18]. Furthermore, it is important to note that this method primarily measures behavior rather than the emotional dimension of empathy.
4. Psychophysiological measures: Psychophysiological measures assess physiological responses linked to empathy, like heart rate changes, skin conductance, and brain activity. These measures offer objective data on participants’ physical reactions during empathic experiences. There are studies [19, 20] examining psychophysiological measures to understand empathy’s neural and physiological mechanisms. They used techniques like fMRI and EEG to explore brain activity during tasks involving empathetic responses. While not predicting empathy directly, their research has enhanced the understanding of neural processes in empathetic experiences, elucidating the role of mirror neurons and brain areas responsible for emotional processing in contributing to this comprehension. However, psychophysiological measures also have their limitations: sensitivity to non-empathetic factors, potential inter-individual variability, and equipment constraints.

The choice of measurement depends on the specific research objectives, population under study, and the dimensions of empathy being assessed. It is important to consider that each measurement method has its strengths and limitations. To combat the limitations, researchers often use multiple measures in combination to capture the different facets of empathy comprehensively. This increases the complexity of measuring empathy, even though some individual measures are already complex and time consuming. Ideally we would like a measure of empathy that is reasonably objective and can be while applied during arbitrary tasks (i.e., it does not require the performance of a specific task). Psychophysiological measures satisfy these requirements best, the precise connection between these measures and empathy remains an open question. Our study aims to aims to bridge this gap by developing a powerful machine learning model that directly measures empathy based on physiological signals, offering a novel and more targeted perspective on empathetic responses.

VR is a computer-generated technology that simulates a realistic environment, allowing users to immerse themselves and interact with the virtual world. By wearing a VR headset and sometimes using accompanying peripherals, users can experience a sense of presence and engage with virtual objects and environments as if they were physically present, providing an immersive and often interactive experience [21, 22]. VR has shown promise in evoking empathy and emotional engagement across various domains and has been explored as a valuable tool for empathy training [23, 24]. Thereby, numerous scientific papers have put forth assertions that VR has the potential to evoke empathic behavior, labeling VR technology as ‘the ultimate empathy machine’ [25]. Some of the key points why VR is called ‘the ultimate empathy machine’ are: 1) immersive and embodied experience: VR can create a sense of presence and immersion, allowing users to embody different perspectives and experiences. This immersive quality has the potential to enhance empathy by enabling individuals to see and feel situations from another person’s point of view [26]; 2) perspective-taking and emotional engagement: VR experiences can simulate realistic scenarios that elicit emotional responses and engage users in perspective-taking. By experiencing the world through someone else’s eyes, individuals may develop a deeper understanding of others’ feelings and experiences [27]. There is a study that demonstrated VR has the potential to enhance emotional well-being and evoke empathy among the elderly. It exposed them to emotionally engaging content, such as storytelling elements and immersive environments depicting scenarios relevant to their interests, like virtual tours of nostalgic places. Additionally, it facilitated simulated social interactions with family members or peers, designed to evoke empathy and emotional engagement [28]; 3) empathy training and perspective shift: In domains like healthcare, education, and diversity training, VR-based interventions are designed to enhance empathic abilities. By immersing individuals in lifelike scenarios, VR challenges their preconceptions and encourages perspective-taking. The highly realistic and interactive nature of VR experiences allows participants to gain deeper insights into the emotions and experiences of others, making it a powerful tool for empathy training [24]. There is a study where VR emerges as a powerful tool for cultivating empathy, particularly in addressing sensitive topics like racism, inequity, and climate change within the medical field. Through immersive VR experiences, participants engage in transformative conversations, enhancing their capacity for empathy and understanding in diverse healthcare contexts [29]. Additionally, there is a study that investigates the impact of dementia VR-based training with peer support on home care workers’ understanding and empathy towards dementia patients. The researchers assessed the effects of a comprehensive training program on dementia knowledge, attitudes, competence, and empathy among home care workers through a cluster randomized controlled trial and the results indicate significant improvements in these domains among participants who received the VR-based training compared to the control group, suggesting the efficacy of innovative training approaches in enhancing dementia care skills among home care workers [30]; 4) ethical considerations: While VR can foster empathy, its use must be approached with ethical considerations. As an ‘ultimate empathy machine,’ VR has the potential to evoke strong emotional responses in users. Thus, responsible design is essential to ensure that VR experiences respect the dignity and privacy of individuals involved. Moreover, careful management of emotional impact is crucial to prevent any potential negative consequences on participants’ well-being. Striking a balance between creating immersive empathic experiences and safeguarding participants’ emotional welfare is imperative in utilizing VR as an empathy-enhancing tool [31].

The aim of this study is to research how the empathy of the participants is related to changes in their physiological attributes measured by different sensors (inertial measurement unit, photoplethysmogram, eye tracking, electromyography (EMG), electrodermal activity, 3-axis accelerometer). Using a VR headset, the participants are going to be immersed in 3-dimensional 360° videos of actors who express different emotions (sadness, happiness, anger and anxiousness). They will report their experience of empathy through short questionnaires. Based on their sensor and questionnaires data, a machine learning model will be developed that will predict a person’s score on an empathy questionnaire based on their physiological arousal while watching VR videos.

The development of a stable and accurate model for predicting people’s level of empathy would offer several benefits as: 1) personalized interventions and early intervention/prevention: individuals’ empathy levels could be assessed in various contexts, such as healthcare, education, and counseling, allowing for tailored interventions and treatments to enhance empathic abilities. Additionally, identifying individuals with lower empathy levels early on could enable targeted interventions and preventive measures, particularly in fields where empathy is crucial, such as healthcare and interpersonal relationships, helping to avert potential negative outcomes or conflicts. 2) selection and training: the model could assist in selecting individuals for roles that require high levels of empathy, such as counseling, customer service, or leadership positions. It could also guide training programs by identifying areas where individuals may benefit from additional support or development; 3) research and understanding: a reliable model for predicting empathy levels could contribute to research efforts in understanding empathy and its role in various aspects of human behavior, relationships, and societal dynamics. It could provide insights into factors influencing empathy and help identify patterns and trends across different populations; 4) entertainment and interactive media: by tailoring content to users’ empathic tendencies, creators can offer more emotionally engaging experiences in video games, interactive narratives, and recommend personalized media choices, enhancing emotional resonance and user satisfaction; 5) personal growth and self-awareness: individuals who have access to information about their own empathy levels could gain a better understanding of their strengths and areas for improvement. This self-awareness could foster personal growth and encourage individuals to develop and cultivate empathy as a valuable skill. Overall, a stable and accurate model for predicting people’s level of empathy could have significant implications for personalized interventions, early intervention and prevention, selection and training processes, research advancements, entertainment and interactive media, and individual self-awareness and growth in fostering empathy [3, 5, 32, 33].

## Materials and methods

### Empathy elicitation using VR

Based on the narrative review on the use of VR for eliciting empathy [1], we have decided to immerse participants in 360º and three-dimensional (3D) virtual environment due to VR’s efficacy in eliciting empathic responses. Our choice of a 360º immersive setting is substantiated by comparative analyses demonstrating that it is equally or more effective at eliciting empathy compared to other approaches such as two-dimensional video/film [1, 34, 35], curriculum contents like workshops [36] or e-course materials [37], narrative-based perspective-taking exercises [9, 38], and text-based information [9, 35]. This effectiveness is observed immediately or with a lasting positive impact. Additionally, our decision to utilize a 3D level of immersion aligns with the findings of the review [1], which highlight the superior effectiveness of 3D immersion over two-dimensional.

During the VR experience, participants will adopt a first-person perspective, allowing them to observe the actors expressing emotions directly in front of them. This approach was chosen because research suggests that a first-person perspective is more effective than a third-person perspective, where the participant merely observes events happening among others [39, 40].

**Fig 1:**
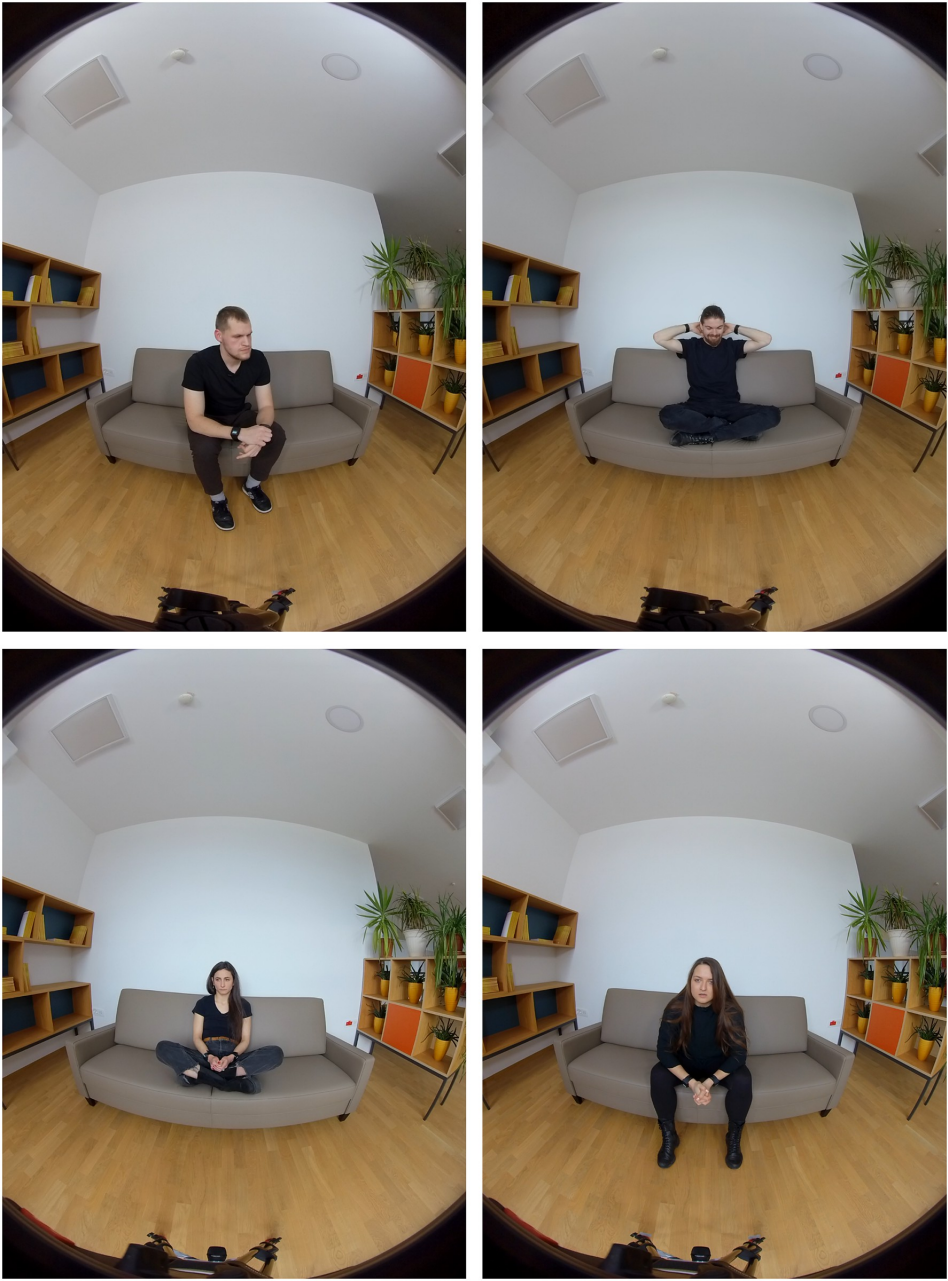
Top to down: (left) Actor one (Dave in the narrative) expressing anger and (right) Actor two (Jake in the narrative) expressing happiness; (left) Actress one (Anna) expressing sadness and (right) Actress two (Leah) expressing anxiousness.

We decided on videos where actors genuinely express four distinct emotions (happiness, sadness, anger, and anxiousness), without any accompanying content, words, or explanations. This decision was driven by the aim to minimize the potential influence of content on evoking emotions, as it would complicate the differentiation between empathy and emotions triggered by the content itself [31]. Moreover, individuals may exhibit distinct empathic responses to different emotional valences, which is why we have included four different emotions in the study [41]. However, recognizing that some participants may find it more impactful to understand the reasons behind the emotions, we also developed a narrative version of the VR session. This version includes an emotional narrative conveyed through audio, followed by a corresponding video of the actor expressing the emotion (50—120 seconds). In order to ensure a gender-balanced distribution, we have recorded videos featuring two male and two female actors. Furthermore, we have created two separate versions of the narrative. Each version is composed of four distinct parts, with each part corresponding to one of the expressed emotions (Fig 1). The narratives uphold a thematic consistency concerning childhood abuse, abandonment, and mother-child relationships.

In the first narrative, we follow the story of two sisters, Leah and Anna, whose childhood was marred by fear and violence due to their abusive father, leading to their mother’s departure. The sisters carry the painful memory of silently watching their mother pack her bags, eventually ending up in an orphanage. Leah finds solace and success in her academic pursuits, securing a scholarship to study abroad. However, a recent encounter with their mother reignites Anna’s anger as she confronts her about the past, only to be met with a lack of remorse. This encounter leaves Anna questioning the concept of family and regretting their decision to reconnect (see S1 Appendix). There is also one more version written for males with the same storyline (see S2 Appendix).

The second narrative delves into the experiences of two brothers, Jake and Dave. Jake learns of their mother’s stroke after three years of no contact, and he is overwhelmed by sadness. He is deeply hurt by her blame and refusal to see them. Meanwhile, Dave grapples with anxiousness, burdened by their dysfunctional family dynamics and regretting his incomplete education. Although he desires a job change, he hesitates to seek assistance, considering the responsibilities his brother already faces. On a positive note, Jake experiences happiness and anticipation as he and his girlfriend expect a baby, triumphing over their concerns about conception. Lastly, Dave expresses his anger towards their parents for the detrimental impact on his life (S3 Appendix). There is also one more version written for females with the same storyline (see S4 Appendix).

### VR sessions

The sessions, conducted with and without narratives, are structured in the following manner:

1. With narratives:
  - In preparation for the session, the participants will be requested to fill out questionnaires pertaining to personal information, including age, gender, health status, proficiency in the Slovenian language, prior experiences with VR, employment or study status, field of work or study, and educational background. This questionnaire, developed by our team, is provided in S5 Appendix. Additionally, participants will be asked to complete the Questionnaire of Cognitive and Affective Empathy (QCAE) [3] to assess their trait empathy. Trait empathy represents the individual’s innate capacity for empathetic behavior and is closely linked to personal characteristics [42].
  - The first step of the VR session is calibration. To calibrate the baseline, participants will be asked to relax, breathe normally, and avoid facial expressions and head movements during a 2-minute recording. They will later be prompted to perform several voluntary facial expressions. The data recorded during these voluntary facial expressions will inform the subject-specific normalization of EMG signals, following the approach suggested in certain studies [43–45].
  - Following the calibration process, participants will be exposed to a 2-minute forest video. Its purpose is to establish a baseline, reflecting the participants’ relaxed state. The forest video utilized in this study was an existing video titled ‘The Amsterdam Forest in Springtime,’ sourced from YouTube.
  - Subsequently, the main segment of the session entails the four sets of narrative-video pairs. Each pair consists of the emotional narrative read by the corresponding actor, followed by the video portraying the actor expressing the specific emotion. The emotions portrayed in these pairs include anger, sadness, happiness, and anxiousness. The duration of each narrative-video combination ranges from 2 to 3 minutes. The participants will be instructed to empathize with the individuals in the video recordings.
  - Following the observation of each narrative-video pair, participants will provide brief feedback on their current empathic state. They will do so by answering three out of the four different items that measure affective empathy from the 12-item State Empathy Scale developed by Lijiang Shen, based on the Interpersonal Reactivity Index (IRI). The selection of these three questions specifically is made because factor analysis requires at least three indicators or items per factor. We have excluded the first item in this scale as it appears to assess the quality of the actor’s performance rather than empathy. Therefore, we specifically utilized the 2nd, 3rd, and 4th items before moving on to the next pair [46]. State empathy refers to the temporary affective response evoked in specific situations [47]. This procedure will be repeated four times, with each iteration featuring a distinct emotion. The duration of each empathic state report is expected to be up to 30 seconds.
  - In addition to the state empathy questions, participants will be requested to provide brief feedback on their current arousal and valence states using the arousal and valence statements from the self-assessment manikin (SAM) affective rating system [48]. This inclusion of arousal and valence measures alongside state empathy is crucial because it offers a more comprehensive insight into emotional experiences during VR sessions. Arousal and valence are fundamental components of emotional responses and are often intertwined with empathy [49].
  - After the participants provided brief feedback on their current arousal and valence states, we included one item to assess personal distress, specifically question number 17 from the personal distress items based on the IRI [15].
  - The final segment of the session involves a roller coaster video titled ‘Official 360 POV—Yukon Striker—Canada’s Wonderland’, which was sourced from YouTube. This video, as a control measure were implemented, to address potential confounding variables, particularly to differentiate between non-empathic psychological arousal and the empathic arousal expected from the main video. While the primary response stimuli consisted of videos designed to evoke empathic physiological arousal, a control stimulus, namely a roller coaster video, was incorporated to induce non-empathic arousal. We believe that this strategic control will facilitate the differentiation between empathic and non-empathic responses, enhancing the validity and reliability of the study’s findings. One of our experiments aims to examine the differences in physiological responses between empathically stimulating content and non-empathic, arousal-inducing content, such as the roller coaster video.
  - The session’s final segment is followed by the arousal and valence statements from the SAM affective rating system [48], with the aim of comparing the intensity of arousal and valence after the roller coaster video and arousal, valence and empathic states following each pair of narrative-video.
  - At the conclusion of the session, we will administer a presence questionnaire adapted from the Bongiovi Report [50]. The questionnaire has been modified to include questions from the presence section, as well as an additional inquiry regarding any challenges encountered by participants that may have hindered their VR experience (refer to S6 Appendix).
2. Without narratives:
  - The overall procedure remains the same as with narratives, with the exception of the main segment where the four emotions will be portrayed by all four actors without accompanying narratives. The actors will exhibit a gradual progression into each emotion, reaching its peak intensity before returning to a neutral state. This session is suitable for participants of all linguistic backgrounds.

Taking into account recommendations for minimizing VR sickness and potential health concerns, the duration of the videos, including the calibration step, will be around 20 minutes [51]. Each participant will be assigned one of five available versions for viewing: actresses with the first narrative version, actresses with the second narrative version, actors with the first narrative version, actors with the second narrative version, or the version without narrative.

### Study design and study population (Recruitment)

Convenience sampling will be employed to recruit participants from the general public. There will be no specific pattern for selecting respondents, as participants will be invited from various sources, including Jožef Stefan Institute employees, students from different university programs, and individuals from the general public. Potential participants will receive verbal or written invitations to participate in the study. Exclusion criteria will include individuals under 18 years of age, those diagnosed with epilepsy or heart conditions, individuals with health conditions or medication use that significantly impact their autonomic nervous system, and individuals with anxiety disorders (generalized anxiety disorder, panic disorder, social anxiety disorder, post-traumatic stress disorder). Inclusion criteria consist of a willingness to participate in scientific research and having normal vision, minor refractive errors, or the use of contact lenses.

We strive for age and gender-balanced groups, ensuring equal representation of male, female, and potentially other gender identities. Regarding education levels and study/work fields, our aim is to include individuals with diverse educational and professional backgrounds. We expect to have a participant pool of over 100 individuals.

Given that the primary objective of this study is to examine the association between changes in physiological attributes and empathy based on individual emotions, it falls under the category of a correlational study. A correlational approach will be employed to explore relationships among the aforementioned variables.

Ethical clearance for this study was obtained from the Research Ethics Committee at the Faculty of Arts, University of Maribor, Slovenia (No. 038–11-146/2023/13FFUM), as the participation of individuals in the research poses no risk of physical, psychological, legal, or social harm. Written informed consent was obtained from the actors prior to recording. Additionally, the individuals in this manuscript have given written informed consent (as outlined in PLOS consent form) to publish these case details. Participants will be informed about the study verbally, with the option to stop their participation at any point. Subsequently, they will receive a separate informed consent form prepared for them to sign prior to their participation in the study.

### Data collection

**Fig 2:**
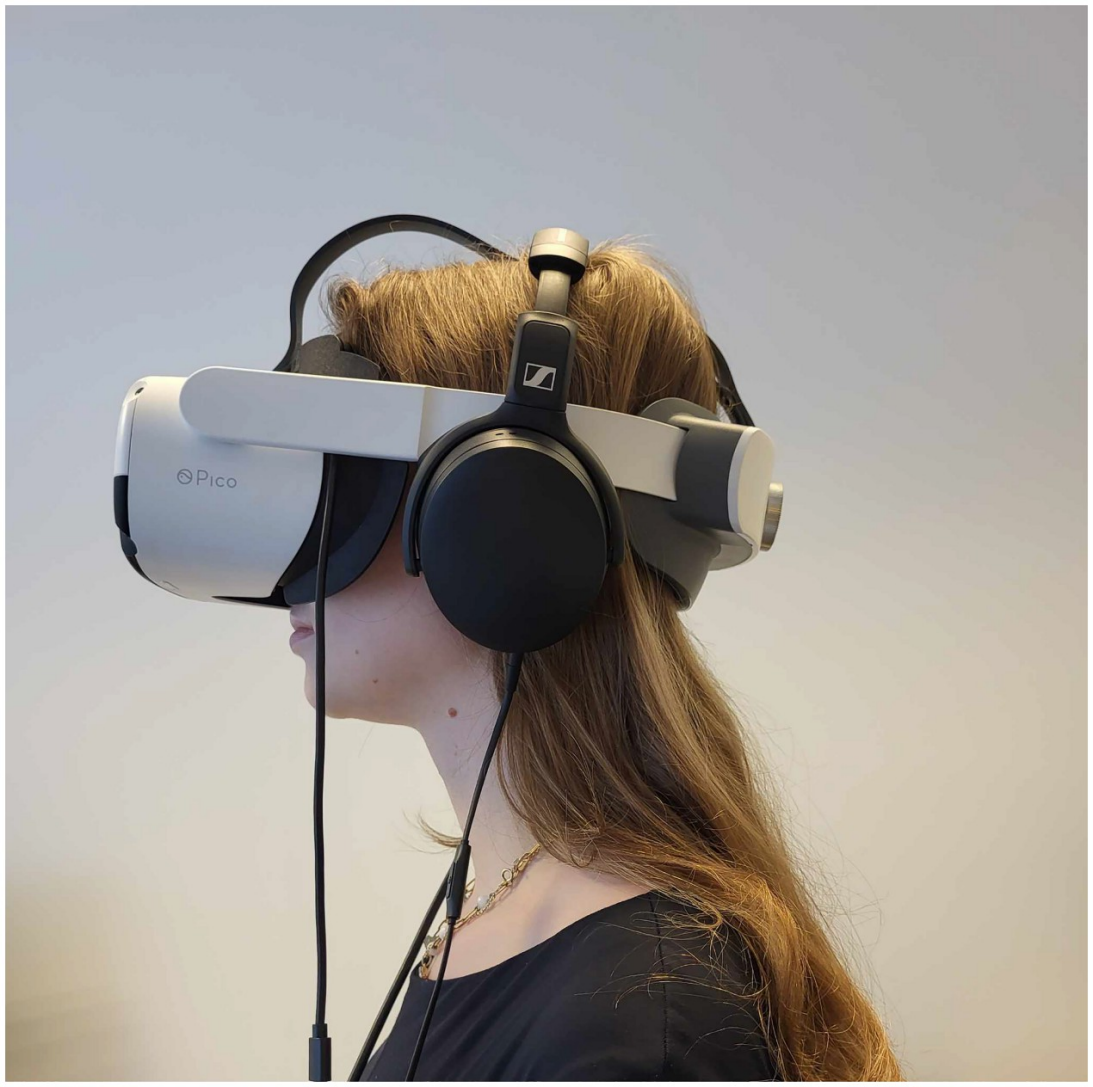
A participant watching videos via Pico Neo 3 Pro Eye headset in the process of collecting the dataset.

Data from the participants will be collected using two devices: the emteqPRO system and the Empatica E4 wristband. The emteqPRO system consists of a sensing mask attached to the Pico Neo 3 Pro Eye VR headset (Fig 2). It includes features such as EMG to measure facial muscle activation (expressivity), photoplethysmography (PPG) for heart-beat monitoring, inertial measurement unit (IMU) to track head motion, and eye tracking to observe eye movements and objects of interest. The Empatica E4 wristband includes an electrodermal activity (EDA) sensor (galvanic skin response), an infrared thermopile that reads peripheral skin temperature, a 3-axis accelerometer that captures motion-based activity and a PPG sensor [43]. Both devices are equipped with an internal clock [52, 53].

In addition to raw sensor data, the EmteqPRO system also provides derived variables. Integral to the emteqPRO system, the Emteq Emotion AI Engine uses data-fusion and machine learning algorithms to analyze the multimodal sensor data and recognize the user’s affective state. Comprising 7 different modules, the Emteq emotion AI engine provides affective insights for each recording. The ‘Data Insights’ feature in the SuperVision application, a web-based signal monitoring application, processes the data and generates a file containing 29 derived features, with 22 of them describing the seven modules [43]:

- 7 features describing heart-rate variability (HRV) derived from PPG;
- 3 features describing breathing rate, derived from PPG [54];
- 2 features describing expressions obtained from EMG;
- 4 features describing arousal, obtained from both EMG and PPG [55];
- 4 features describing valence, obtained from both EMG and PPG [55];
- 1 feature describing facial activation that is determined through EMG;
- 1 feature describing facial valence that is assessed using facial muscle data obtained through EMG.

Moreover, except the mentioned modules and the 22 features describing them, head activity is included as a feature, which indicates the percentage of the recording session during which head movement is detected. Additionally, dry EMG electrodes are strategically positioned over the zygomatic, corrugator, frontalis, and orbicularis muscles, which play a pivotal role in evaluating objective valence responses to stimuli. The EMG data hence yields four additional features, each delineating the activation of the corresponding muscles detected by their respective sensors. This activation is expressed as a percentage of the maximum activation observed during the calibration session.

The Empatica E4 wristband shares similarities with the emteqPRO system, as mentioned earlier, particularly in terms of its PPG sensor. Furthermore, it also provides derived variables, too. Thus, we will extract the following data from the Empatica E4 wristband (17):

- blood volume pulse (BVP) obtained from the PPG signal;
- average heart rate extracted from the BVP signal;
- fluctuations in specific electrical properties of the skin, gained from the EDA sensor;
- motion-based activity obtained from the 3-axis accelerometer;
- peripheral skin temperature, captured through the infrared thermopile;
- time between individual heart beats extracted from the BVP signal.

In addition to the sensor data, our analysis will incorporate features obtained from the mentioned questionnaires. These include the personal information questionnaire, the trait empathy questionnaire, the presence questionnaire, the arousal and valence questions after the roller coaster video, and the state empathy, arousal and valence questions asked four times, because the participants will be instructed to regularly report their empathy towards the actors after each emotion expressed in the video session.

There are no established sensor measures of empathy, but we expect that sensors suitable for measuring other emotional states will be appropriate for empathy as well. We will use a wide range of sensors to understand the connection between participants’ empathic responses and the emotions expressed by actors in 3-dimensional 360° videos. Empathy, defined as the ability to comprehend and share the emotional states of others, is actually an emotion that can be mirrored in participants’ reactions as they immerse themselves in emotional scenarios. Our selected sensors are chosen to capture physiological responses corresponding to specific emotions: 1) when participants experience sadness, their gaze patterns, as evidenced by studies on the connection between music, emotion, and gaze [56], tend to be more focused or prolonged on stimuli associated with sadness. Facial expressions related to sadness, such as a downturned mouth or raised inner eyebrows, can be detected through EMG [57]. Additionally, EDA may register an increase in skin conductance due to the emotional arousal induced by sadness [58]; 2) in moments of happiness, dynamic and responsive gaze patterns to positively valenced stimuli [59] can be observed through eye tracking. Similarly, EMG can capture facial expressions associated with happiness, like smiling and muscle activities around the eyes [60]; 3) anxiety, marked by erratic gaze patterns or heightened focus on threat-related stimuli, can be discerned through eye tracking. Studies exploring the link between pupil size and emotional arousal provide additional insights [61]. Facial expressions related to anxiety, such as widened eyes or a tense jaw, can be monitored using EMG. EDA may register an increase in skin conductance due to heightened emotional arousal and stress [62]; 4) lastly, in instances of anger, gaze patterns may focus on stimuli related to the source of anger, and studies on attention to angry faces support this observation [63]. EMG can capture facial expressions related to anger, including a furrowed brow and clenched jaw [57]. Through this multi-sensor approach, we aim to decode the complex connection between physiological attributes and empathic responses.

The entire dataset will be securely stored on the computer servers of the research institute in Slovenia (Jožef Stefan Institute). To ensure participants’ privacy, our team will implement access control measures. The study data will only be accessible to authorized users via the local internet connection or through a Virtual Private Network (VPN). Furthermore, all data will be pseudonymized by replacing participants’ full names with a unique participant ID—a randomly generated numeric identifier. The link between the participant ID and their actual identity will be stored separately from all other data, ensuring an additional layer of data protection. Upon participants’ request, their data will be deleted, and the data will be fully anonymized upon publication, i.e., the link between the random ID and their identity will be destroyed.

### Software used

The videos for empathy elicitation were recorded using a professional 8K 3D VR camera Insta360 Pro. Subsequently, we utilized the ‘insta360 stitcher’ software, a free tool from Insta360 designed specifically for stitching footage captured by their Pro camera. Stitching involves merging multiple views of the same video to create a seamless whole. For the purpose of video editing, where we needed to combine different videos into a single cohesive piece, we employed ‘Kdenlive’, an open-source cross-platform video editing program capable of handling videos larger than 4K. To edit the narrative audio and audio from the videos, we turned to ‘Adobe Premiere Pro’.

To establish the connection between the VR headset and the computer, we utilize ‘SteamVR’, which provides the necessary components for Pico Neo 3 Pro Eye headset functionality. Additionally, we employ ‘Pico DisplayPort Assistant’ and ‘Streaming Assistant’ to facilitate the same. To play back the videos to the participants, we use the ‘Whirligig’ media player application, which supports special codecs. For recording participants’ sensor data and performing calibration, we rely on the ‘Emteq SDK’, while ‘Emteq DAB tools’ serves as a tool for accessing and reviewing results without the need to send them to the Emteq Emotion AI Engine. ‘Openface’ is used for real-time data observation, data retrieval, and transmission to the Emteq Emotion AI Engine for further processing. As for the data obtained from the Empatica E4 wristband, we access, review, and manage it through their cloud-based repository website.

### Data analysis

In order to process the collected data effectively, several steps will be taken. Firstly, the derived features obtained from each module of the Emteq Emotion AI Engine, as well as the features from the Empatica E4, will be combined by concatenating the data files. The epoch timestamps will then be converted to human-readable date and time formats, and the output labels will be populated based on the scores from the empathy questionnaires. The classification task will involve multi-label classification, with the affective component of trait empathy and state empathy serving as the labels of interest.

If a variable has a modest amount of missing values, imputation will be used. Otherwise, if the amount is substantial, the corresponding data will be dropped. The derived features are provided by different devices with data updated at different intervals, ranging from 10 seconds to 500 milliseconds, resulting in varying data frequencies. Undersampling or oversampling will be used to handle the high or the low frequency of the derived features accordingly, and they will be aligned at a consistent interval. Regarding the variables, while some are ready to be used, as the derived features are, from others, such as the raw sensor data, we will extract features.

The research aims to address several key questions. Firstly, it seeks to explore the connection between physiological attributes and empathy. For this purpose we will use multiple standard machine learning classifiers to develop prediction models, with different parameters tested to optimize performance. The predictive models will be developed based on the descriptive space consisting of physiological data from the sensors and data from the questionnaires. Additionally, personalized models will be explored to enhance predictions. Personalized feature values will be utilized, employing normalization techniques by calculating the ratio between the parameter value and the average value for each participant over the entire viewing period. If the initial results prove unsatisfactory, deep learning techniques will be applied directly to the raw sensor data, bypassing the use of the derived features provided by Emteq. Furthermore, we will explore deep learning approaches using the derived features, allowing us to consider both options.

Moreover, the research will examine potential gender differences in empathy, specifically exploring whether female participants display higher levels of empathy compared to males [64, 65]. For that purpose, we will conduct a t-test [66]. In terms of the material presented to participants, an experiment will be conducted to determine if individuals respond more intensely to videos with narratives, where the emotional context is known, or to videos showcasing expressions without contextual information. This experiment aims to verify whether participants’ knowledge of the narrative as prior to the videos enhances their empathic responses. Previous research has suggested that exposure to narrative-based perspective-taking exercises after watching a 360° video can lead to significantly higher empathy scores [38]. For this purpose, we will use the t-test as well. Additionally, the study aims to investigate whether certain emotions elicit stronger reactions from individuals compared to others, utilizing an ANOVA test [67]. Lastly, we are interested in exploring how different emotions are related to physiological responses, for which we will use a MANOVA test [68]. Multivariate analysis of variance (MANOVA)).

In addition to our primary objectives, our comparison will involve two types of videos where actors express the same four emotions. While assessing the effectiveness of different narratives themselves falls outside the scope of our investigation, because this evaluation would pertain not to the emotions being conveyed but rather to our proficiency in crafting stories, we can nonetheless discern, using the ANOVA test, which one carries more impact. This insight could potentially prove valuable for future applications.

The study will not specifically consider which pair of actors (female or male) elicits stronger responses from participants. This is because the actors’ individual acting skills may influence participants’ reactions, which is not within the scope of the research focus.

By addressing these questions, the study aims to deepen our understanding of the relationship between physiological attributes, emotions, gender differences in empathy, and the impact of narrative context on empathic responses, providing valuable derived features into the complex dynamics of emotional experiences.

## Discussion

Understanding and measuring empathy has broad implications, impacting mental health interventions, VR design, and empathy training. Measuring empathy deficits in psychiatric disorders with models such as we plan to build can be used to initiate, monitor, and adapt therapeutic interventions. VR, as the “ultimate empathy machine,” can transform empathy training in healthcare, education, and other contexts by immersing individuals in lifelike scenarios, and our models can again be used for monitoring and guiding the training. This way, it can more successfully challenge preconceptions, foster perspective-taking, and enhance empathic abilities. The ability to measure empathy can also be used in entertainment, to adapt VR content so that the user experiences it more fully, develops stronger (or weaker, as desired) feelings for virtual characters, and similar. All this can also contribute to personal growth and self-awareness, emphasizing empathy as a valuable skill.

This research endeavors to establish a “golden standard” for measuring empathy as a primary outcome. Additionally, it aims to differentiate the impact of various emotions on physiological attributes, identify the emotion that elicits the most intense empathic response from participants, explore potential gender differences in the intensity and general nature of responses, and investigate whether emotions expressed with narratives or without content exert a stronger influence on empathic responses.

### Strengths and limitations

Through this research, we aim to gain a comprehensive understanding of the potential outcomes and the potential directions that further research in this field could take. However, it is important to recognize specific limitations in addition to the potential listed findings. The sensors we will use are not feasible for everyday use, and consequently neither can the method for measuring empathy we plan to develop. Nevertheless, it can be used in VR, and measuring empathy in VR is particularly valuable since VR technology is often referred to as the ‘ultimate empathy machine.’ One limitation pertains to the study’s suitability for laboratory conduction rather than real-world investigation. As a result, the measures obtained may not fully represent naturalistic empathic responses.

There remains a possibility that participants exposed to the narrative versions may be influenced by the specific storyline. Similarly, participants watching the non-narrative versions could also be influenced by the actors’ portrayals, potentially limiting the study’s generalizability. To mitigate this potential influence from the particular scenario, variations in the narratives have been introduced. Additionally, an extra version without narratives has been included. This indicates our conscientious effort to evoke empathy in a highly realistic manner and devise the most comprehensive procedure feasible.

While our intent is to deeply immerse participants and mitigate factors that could disrupt this immersion, certain considerations must be made. Specifically, the presence of facial sensors on the VR headset leads to the exclusion of participants who wear glasses. Consequently, our method potentially limits the inclusion of older individuals in the sample. Since the study is expected to primarily involve a younger population aged 18 and above, there might be physiological attribute variations and distinct responses to empathy scenarios among older individuals that will not be adequately represented in our sample size. As a result, the study’s scope may be limited concerning the generalization of findings to the broader population, particularly in regards to older age groups.

In statistical modeling, established methodologies exist to calculate the required sample size for achieving model stability. However, in the context of utilizing machine learning models, it is challenging to precisely determine the optimal sample size. For our study, we anticipate that approximately 100 participants will suffice to develop a stable predictive model. While we cannot determine this with certainty beforehand due to the complexities of machine learning algorithms, it is worth noting that previous studies [34, 37–40, 69] have successfully developed stable models using even smaller sample sizes. Based on this precedent, we believe that our chosen sample size will be adequate for the purposes of our investigation.

While convenient for logistical reasons, our use of convenience sampling introduces potential limitations. In employing this method, our sample may not be fully representative of the broader population due to potential biases associated with self-selection. This could limit the generalizability of our findings beyond the specific group of individuals who volunteered or were readily available. Nevertheless, the practicality of this approach expedites participant recruitment, allowing for extensive data collection in a controlled laboratory setting. Despite acknowledged limitations, we believe our targeted investigation will yield valuable insights into the relationships between empathy, physiological attributes, and immersive VR experiences within the context of our specific sample.

One of the primary challenges encountered in studies of this nature lies in establishing a reliable ground truth. We plan to do this with questionnaires. However, it is important to acknowledge that participants grading their own empathic states may introduce errors in the form of subjective biases. In light of this consideration, we will use two distinct questionnaires, one for measuring state empathy and another for trait empathy. This approach aims to minimize potential errors in our measurements in a way that may reveal some pattern of connections between the two.

### Dissemination plans

Following data collection, we intend to publish a comprehensive paper encompassing the dataset analysis and the development of predictive models. Additionally, we plan to make the dataset publicly available, along with the data analysis outcomes and the 3D and 360° videos. This dataset is a valuable resource for research on empathy, as there is currently a lack of similar datasets and videos available. Our plans comply with ethical considerations, as we have obtained signed informed consents from the actors involved in the video recordings and we are going to obtain informed consent for sharing the data from each participant. The dataset will be appropriately anonymized, disallowing any direct connection to the individual participants’ identities, thus safeguarding their privacy.

## Supporting information

S1 Appendix Narrative 1. Female version: Leah and Anna.
(PDF)

S2 Appendix Narrative 1. Male version: Andrew and Dominic.
(PDF)

S3 Appendix Narrative 2. Male version: Jake and Dave.
(PDF)

S4 Appendix Narrative 2. Female version: Laura and Kate.
(PDF)

S5 Appendix Personal information questionnaire. (PDF)

S6 Appendix Presence questionnaire. (PDF)

## References

[1] Kizhevska E., Ferreira-Brito F., Guerreiro T., Luštrek M. (2022). Using Virtual Reality to elicit Empathy: a narrative review.

[2] Harari H., Shamay-Tsoory S. G., Ravid M., Levkovitz Y. (2010). Double dissociation between cognitive and affective empathy in borderline personality disorder. Psychiatry research, 175(3), 277–279. doi: 10.1016/j.psychres.2009.03.002 20045198

[3] Reniers R. L., Corcoran R., Drake R., Shryane N. M., Völlm B. A. (2011). The QCAE: A questionnaire of cognitive and affective empathy. Journal of Personality Assessment, 93(1), 84–95. doi: 10.1080/00223891.2010.528484 21184334

[4] Decety J., Jackson P. L. (2006). A social-neuroscience perspective on empathy. Current Directions in Psychological Science, 15(2), 54–58. doi: 10.1111/j.0963-7214.2006.00406.x

[5] Shamay-Tsoory S. G., Aharon-Peretz J. (2007). Dissociable prefrontal networks for cognitive and affective theory of mind: a lesion study. Neuropsychologia, 45(13), 3054–3067. doi: 10.1016/j.neuropsychologia.2007.05.021 17640690

[6] Han S., 2015. Understanding cultural differences in human behavior: a cultural neuroscience approach. Current opinion in behavioral sciences, 3, pp.68–72. doi: 10.1016/j.cobeha.2015.01.013

[7] Batson C. D. (2011). Altruism in humans. Oxford University Press.

[8] Ma-Kellams C., Lerner J. (2016). Trust your gut or think carefully? Examining whether an intuitive, versus a systematic, mode of thought produces greater empathic accuracy. Journal of personality and social psychology, 111(5), 674. doi: 10.1037/pspi0000063 27442764

[9] Herrera F., Bailenson J., Weisz E., Ogle E., Zaki J. (2018). Building long-term empathy: A large-scale comparison of traditional and virtual reality perspective-taking. PloS one, 13(10), e0204494. doi: 10.1371/journal.pone.0204494 30332407 PMC6192572

[10] Eisenberg N., Spinrad T. L., Sadovsky A. (2006). Empathy-related responding in children. In Killen M. Smetana J. G. (Eds.), Handbook of moral development (pp. 517–549). Psychology Press.

[11] Eisenberg N., Fabes R. A. (1998). Prosocial development. In Damon W. (Series Ed.), Eisenberg N. (Vol. Ed.), Handbook of child psychology: Vol. 3. Social, emotional, and personality development (5th ed., pp. 701–778). Wiley.

[12] Eisenberg N., Miller P. A. (1987). The relation of empathy to prosocial and related behaviors. Psychological Bulletin, 101(1), 91–119. doi: 10.1037/0033-2909.101.1.91 3562705

[13] Hoffman M. L. (2000). Empathy and moral development: Implications for caring and justice. Cambridge University Press.

[14] Lima F. F. D., Osório F. D. L. (2021). Empathy: assessment instruments and psychometric quality–a systematic literature review with a meta-analysis of the past ten years. Frontiers in psychology, 12, 781346. doi: 10.3389/fpsyg.2021.781346 34899531 PMC8653810

[15] Davis M. H. (1980). A multidimensional approach to individual differences in empathy.

[16] Baron-Cohen S., Wheelwright S. (2004). The empathy quotient: an investigation of adults with Asperger syndrome or high functioning autism, and normal sex differences. Journal of autism and developmental disorders, 34, 163–175. doi: 10.1023/B:JADD.0000022607.19833.00 15162935

[17] Lamm C., Batson C. D., Decety J. (2007). The neural substrate of human empathy: effects of perspective-taking and cognitive appraisal. Journal of cognitive neuroscience, 19(1), 42–58. doi: 10.1162/jocn.2007.19.1.42 17214562

[18] Preston S. D., De Waal F. B. (2002). Empathy: Its ultimate and proximate bases. Behavioral and brain sciences, 25(1), 1–20. doi: 10.1017/S0140525X02000018 12625087

[19] Singer T., Lamm C. (2009). The social neuroscience of empathy. Annals of the New York Academy of Sciences, 1156(1), 81–96. doi: 10.1111/j.1749-6632.2009.04418.x 19338504

[20] Keysers C., Gazzola V. (2007). Integrating simulation and theory of mind: from self to social cognition. Trends in cognitive sciences, 11(5), 194–196. doi: 10.1016/j.tics.2007.02.002 17344090

[21] Ahn S. J., Bostick J., Ogle E., Nowak K. L., McGillicuddy K. T., Bailenson J. N. (2016). Experiencing nature: Embodying animals in immersive virtual environments increases inclusion of nature in self and involvement with nature. Journal of Computer-Mediated Communication, 21(6), 399–419. doi: 10.1111/jcc4.12173

[22] Sanchez-Vives M. V., Slater M. (2005). From presence to consciousness through virtual reality. Nature reviews neuroscience, 6(4), 332–339. doi: 10.1038/nrn1651 15803164

[23] Mado M., Herrera F., Nowak K., Bailenson J. (2021). Effect of virtual reality perspective-taking on related and unrelated contexts. Cyberpsychology, Behavior, and Social Networking, 24(12), 839–845. doi: 10.1089/cyber.2020.0802 34129372

[24] Banakou D., Hanumanthu P. D., Slater M. (2016). Virtual embodiment of white people in a black virtual body leads to a sustained reduction in their implicit racial bias. Frontiers in human neuroscience, 601. 10.3389/fnhum.2016.00601 PMC5126081 27965555

[25] Barbot B., Kaufman J. C. (2020). What makes immersive virtual reality the ultimate empathy machine? Discerning the underlying mechanisms of change. Computers in Human Behavior, 111, 106431. doi: 10.1016/j.chb.2020.106431

[26] Riva G., Waterworth J. A., Waterworth E. L. (2004). The layers of presence: a bio-cultural approach to understanding presence in natural and mediated environments. CyberPsychology and Behavior, 7(4), 402–416. doi: 10.1089/cpb.2004.7.402 15331027

[27] Slater M., Antley A., Davison A., Swapp D., Guger C., Barker C., et al. (2006). A virtual reprise of the Stanley Milgram obedience experiments. PloS one, 1(1), e39. doi: 10.1371/journal.pone.0000039 17183667 PMC1762398

[28] Benoit M., Guerchouche R., Petit P. D., Chapoulie E., Manera V., Chaurasia G., et al. (2015). Is it possible to use highly realistic virtual reality in the elderly? A feasibility study with image-based rendering. Neuropsychiatric disease and treatment, 557–563. doi: 10.2147/NDT.S73179 25834437 PMC4357614

[29] Roswell R. O., Cogburn C. D., Tocco J., Martinez J., Bangeranye C., Bailenson, et al. (2020). Cultivating empathy through virtual reality: advancing conversations about racism, inequity, and climate in medicine. Academic Medicine, 95(12), 1882–1886. doi: 10.1097/ACM.0000000000003615 32701556

[30] Sung H. C., Su H. F., Lee W. L., Yamakawa M., Wang H. M. (2022). Effects of a dementia virtual reality‐based training with peer support for home care workers: A cluster randomized controlled trial. International Journal of Geriatric Psychiatry, 37(9). doi: 10.1002/gps.5799 35996760

[31] Bertrand P., Guegan J., Robieux L., McCall C. A., Zenasni F. (2018). Learning empathy through virtual reality: multiple strategies for training empathy-related abilities using body ownership illusions in embodied virtual reality. Frontiers in Robotics and AI, 26. 33500913 10.3389/frobt.2018.00026 PMC7805971

[32] Mehrabian A., Epstein N. (1972). A measure of emotional empathy. Journal of Personality, 40(4), 525–543. doi: 10.1111/j.1467-6494.1972.tb00078.x 4642390

[33] O’Brien E., Konrath S. H., Gruhn D., Hagen A. L. (2013). Empathic concern and perspective taking: Linear and quadratic effects of age across the adult life span. The Journals of Gerontology Series B: Psychological Sciences and Social Sciences, 68(2), 168–175. doi: 10.1093/geronb/gbs055 22865821

[34] Schutte N. S., Stilinović E. J. (2017). Facilitating empathy through virtual reality. Motivation and emotion, 41, 708–712. doi: 10.1007/s11031-017-9641-7

[35] Nelson K. M., Anggraini E., Schlüter A. (2020). Virtual reality as a tool for environmental conservation and fundraising. Plos one, 15(4), e0223631. doi: 10.1371/journal.pone.0223631 32251442 PMC7135095

[36] Stargatt J., Bhar S., Petrovich T., Bhowmik J., Sykes D., Burns K. (2021). The effects of virtual reality-based education on empathy and understanding of the physical environment for dementia care workers in australia: a controlled study. Journal of Alzheimer’s Disease, 84(3), 1247–1257. doi: 10.3233/JAD-210723 34633323

[37] Wijma E. M., Veerbeek M. A., Prins M., Pot A. M., Willemse B. M. (2018). A virtual reality intervention to improve the understanding and empathy for people with dementia in informal caregivers: results of a pilot study. Aging and mental health, 22(9), 1121–1129. doi: 10.1080/13607863.2017.1348470 28691861

[38] Ventura S., Cardenas G., Miragall M., Riva G., Baños R. (2021). How does it feel to be a woman victim of sexual harassment? The effect of 360-video-based virtual reality on empathy and related variables. Cyberpsychology, Behavior, and Social Networking, 24(4), 258–266. doi: 10.1089/cyber.2020.0209 33085513

[39] Fusaro M., Tieri G., Aglioti S. M. (2016). Seeing pain and pleasure on self and others: behavioral and psychophysiological reactivity in immersive virtual reality. Journal of Neurophysiology, 116(6), 2656–2662. doi: 10.1152/jn.00489.2016 27655965 PMC5133301

[40] Fusaro M., Tieri G., Aglioti S. M. (2019). Influence of cognitive stance and physical perspective on subjective and autonomic reactivity to observed pain and pleasure: An immersive virtual reality study. Consciousness and cognition, 67, 86–97. doi: 10.1016/j.concog.2018.11.010 30553938

[41] Olderbak S., Sassenrath C., Keller J., Wilhelm O. (2014). An emotion-differentiated perspective on empathy with the emotion specific empathy questionnaire. Frontiers in Psychology, 5, 653. doi: 10.3389/fpsyg.2014.00653 25071632 PMC4076670

[42] Lawrence E. J., Shaw P., Baker D., Baron-Cohen S., David A. S. (2004). Measuring empathy: reliability and validity of the Empathy Quotient. Psychological medicine, 34(5), 911–920. doi: 10.1017/S0033291703001624 15500311

[43] Gnacek M, Broulidakis J, Mavridou I, Fatoorechi M, Seiss E, Kostoulas T, et al. emteqpro—fully integrated biometric sensing array for non-invasive biomedical research in virtual reality. Frontiers in virtual reality. 2022 Mar 11;3:781218. doi: 10.3389/frvir.2022.781218

[44] Van Boxtel A. (2010, August). Facial EMG as a tool for inferring affective states. Proceedings of measuring behavior (Vol. 2, No. 1, pp. 104–108).

[45] Fridlund A. J., Schwartz G. E., Fowler S. C. (1984). Pattern recognition of self‐reported emotional state from multiple‐site facial EMG activity during affective imagery. Psychophysiology, 21(6), 622–637. doi: 10.1111/j.1469-8986.1984.tb00249.x 6514939

[46] Shen L. (2010). On a scale of state empathy during message processing. Western Journal of Communication, 74(5), 504–524. doi: 10.1080/10570314.2010.512278

[47] Van der Graaff J., Meeus W., de Wied M., van Boxtel A., van Lier P. A., Koot H. M., et al (2016). Motor, affective and cognitive empathy in adolescence: Interrelations between facial electromyography and self-reported trait and state measures. Cognition and emotion, 30(4), 745–761. doi: 10.1080/02699931.2015.1027665 25864486

[48] Bradley M. M., and Lang P. J. (1994). Measuring emotion: the self-assessment manikin and the semantic differential. Journal of behavior therapy and experimental psychiatry, 25(1), 49–59. doi: 10.1016/0005-7916(94)90063-9 7962581

[49] Russell J. A., Barrett L. F. (1999). Core affect, prototypical emotional episodes, and other things called emotion: Dissecting the elephant. Journal of Personality and Social Psychology, 76(5), 805–819. 10.1037/0022-3514.76.5.805 10353204

[50] Title of Project: Virtual Reality study investigating the affective and behavioural impact of enhanced audio-acoustics (Bongiovi) Sussex Innovation Centre, Science Park Square, Brighton BN1 9SB

[51] Vitale S., Sperduto R. D., Ferris F. L. (2009). Increased prevalence of myopia in the United States between 1971-1972 and 1999-2004. Archives of ophthalmology, 127(12), 1632–1639. doi: 10.1001/archophthalmol.2009.303 20008719

[52] Cacioppo J. T., Martzke J. S., Petty R. E., Tassinary L. G. (1988). Specific forms of facial EMG response index emotions during an interview: From Darwin to the continuous flow hypothesis of affect-laden information processing. Journal of personality and social psychology, 54(4), 592. Chicago doi: 10.1037/0022-3514.54.4.592 3367281

[53] Magnée M. J., De Gelder B., Van Engeland H., Kemner C. (2007). Facial electromyographic responses to emotional information from faces and voices in individuals with pervasive developmental disorder. Journal of Child Psychology and Psychiatry, 48(11), 1122–1130. doi: 10.1111/j.1469-7610.2007.01779.x 17995488

[54] Stankoski S., Kiprijanovska I., Mavridou I., Nduka C., Gjoreski H., Gjoreski M. (2022). Breathing rate estimation from head-worn photoplethysmography sensor data using machine learning. Sensors, 22(6), 2079. doi: 10.3390/s22062079 35336250 PMC8951087

[55] Gjoreski M., Kiprijanovska I., Stankoski S., Mavridou I., Broulidakis M. J., Gjoreski H., et al. (2022). Facial EMG sensing for monitoring affect using a wearable device. Scientific Reports, 12(1), 16876. doi: 10.1038/s41598-022-21456-1 36207524 PMC9542454

[56] Krumhansl C. L. (2002). Music: A link between cognition and emotion. Current directions in psychological science, 11(2), 45–50. doi: 10.1111/1467-8721.00165

[57] Ekman P., Friesen W. V. (1978). Facial action coding system. Environmental Psychology and Nonverbal Behavior.

[58] Dawson M. E., Schell A. M., Filion D. L. (2007). The electrodermal system. Handbook of psychophysiology, 2, 200–223.

[59] Hess E. H., Polt J. M. (1960). Pupil size as related to interest value of visual stimuli. Science, 132(3423), 349–350. doi: 10.1126/science.132.3423.349 14401489

[60] Fridlund A. J. (2014). Human facial expression: An evolutionary view. Academic press.

[61] Bradley M. M., Miccoli L., Escrig M. A., Lang P. J. (2008). The pupil as a measure of emotional arousal and autonomic activation. Psychophysiology, 45(4), 602–607. doi: 10.1111/j.1469-8986.2008.00654.x 18282202 PMC3612940

[62] Boucsein W. (2012). Electrodermal activity. Springer Science and Business Media.

[63] Öhman A., Flykt A., Esteves F. (2001). Emotion drives attention: detecting the snake in the grass. Journal of experimental psychology: general, 130(3), 466. doi: 10.1037/0096-3445.130.3.466 11561921

[64] Christov-Moore L., Simpson E. A., Coudé G., Grigaityte K., Iacoboni M., Ferrari P. F. (2014). Empathy: Gender effects in brain and behavior. Neuroscience and biobehavioral reviews, 46, 604–627. doi: 10.1016/j.neubiorev.2014.09.001 25236781 PMC5110041

[65] Schulte-Rüther M., Markowitsch H. J., Shah N. J., Fink G. R., Piefke M. (2008). Gender differences in brain networks supporting empathy. Neuroimage, 42(1), 393–403. doi: 10.1016/j.neuroimage.2008.04.180 18514546

[66] Kim T. K. (2015). T test as a parametric statistic. Korean journal of anesthesiology, 68(6), 540–546. doi: 10.4097/kjae.2015.68.6.540 26634076 PMC4667138

[67] Cuevas A., Febrero M., Fraiman R. (2004). An anova test for functional data. Computational statistics and data analysis, 47(1), 111–122. doi: 10.1016/j.csda.2003.10.021

[68] French A., Macedo M., Poulsen J., Waterson T., Yu A. (2008). Multivariate analysis of variance (MANOVA).

[69] Bouchard S., Bernier F., Boivin E., Dumoulin S., Laforest M., Guitard T., et al (2013). Empathy toward virtual humans depicting a known or unknown person expressing pain. Cyberpsychology, Behavior, and Social Networking, 16(1), 61–71. Chicago doi: 10.1089/cyber.2012.1571 23320872

