# Supplementary material for: Protocol of the study for predicting empathy during VR sessions using sensor data and machine learning
Source: PLoS One. 2024 Jul 18;19(7):e0307385. doi: 10.1371/journal.pone.0307385 (PMC11257359; doi:10.1371/journal.pone.0307385)
Supplement: S1 Appendix — Female version: Leah and Anna. (PDF) [file pone.0307385.s001.pdf]

# APPENDIX A

## NARRATIVE 1

### FEMALE VERSION: LEAH AND ANNA

<u>LEAH - SADNESS</u>

How would I describe my childhood? Oh … not good, really. Brandon – that's my father's name, because I can't call him "dad" after all of this … – he was very irritable and violent. Anna and I were always afraid of him. Towards the end he and mom constantly argued and … he even hit her many times. I still remember the day, when ... when there were bags in the hall. Anna and I stayed in our room and just watched mom through the gap in the door as she packed. I think Anna cried, I didn't. But something inside me broke right then. Somewhere, I don't know, in the stomach. We did nothing, we just let her go. Then we had to live with Brandon for three months, until we were taken from him and put in some kind of an orphanage. Soon he realized he didn't have to pay for our stay if he disowned us. For him, this was easier. All the holidays, all the birthdays … everything was in an orphanage from then on. Other children were going out to be with relatives and such, but not us. This period was difficult for me. I don't like remembering it.

<u>ANNA - ANXIOUSNESS</u>

Uh … Tomorrow at half-past one Leah and I are meeting our mother. I just don't know ... I don't know what to expect, really. Leah wants this, she found her – I don't even know how you can find a person after twenty years, but she did … And now we have to … have a coffee with her, or something, I guess. What do you even say to a person who just packed up and left? This won't be good, this won't be good at all … or maybe it will be? Maybe it will be great. Maybe she has a good reason and is sorry … But if she was sorry, she would contact us herself, right? She must have known where they put us. I don't know, damn, how do you even prepare for a meeting like this? Leah is so excited, but I think she will get hurt. And so will I, but I still have to be there, I guess.

<u>LEAH - HAPPINESS</u>

Okay, so about this letter I've brought with me. Now I can finally say I managed to turn things around for the better. Luckily, I shut the past out and focused on my education. In high school, I gradually started improving, and now in college, my grades are actually high. I received a scholarship for studying abroad, which was amazing for me; I never thought I could achieve it. So, I applied to a few universities that are like, really high-quality. Before, I didn't dare to dream about something like that. And this morning, I received this letter! And you know what? I got accepted!

<u>ANNA - ANGER</u>

Well, yesterday we met with our mother. It was just as I expected, the woman did not even apologize! And we told her everything! What happened to us after she left, how Brandon beat Leah so badly she bled from her ear, how they took us and placed us in that rotten orphanage, so we didn't even see the sea until I was fifteen, how we couldn't even have one normal moment in our childhood, all because of her. But no, she sat there completely cool and then had the balls to say that leaving was the best decision of her life! Excuse me? How can you say that to your daughters?! And then she even had the nerve to ask Leah for money when she found out about her scholarship. Is she even sane? I regret falling for this, having to meet the family, yeah. I've had enough!
