# Supplementary material for: Protocol of the study for predicting empathy during VR sessions using sensor data and machine learning
Source: PLoS One. 2024 Jul 18;19(7):e0307385. doi: 10.1371/journal.pone.0307385 (PMC11257359; doi:10.1371/journal.pone.0307385)
Supplement: S2 Appendix — Male version: Andrew and Dominic. (PDF) [file pone.0307385.s002.pdf]

APPENDIX A1

NARRATIVE 1

MALE VERSION: ANDREW AND DOMINIC

ANDREW - SADNESS

How would I describe my childhood? Oh … not good, really. Brandon – that's my father's name, because I can't call him "dad" after all of this … – he was very irritable and violent. Dominic and I were always afraid of him. Towards the end he and mom constantly argued and … he even hit her many times. I still remember the day, when ... when there were bags in the hall. Dominic and I stayed in our room and just watched mom through the gap in the door as she packed. I think Dominic cried, I didn't. But something inside me broke right then. Somewhere, I don't know, in the stomach. We did nothing, we just let her go. Then we had to live with Brandon for three months, until we were taken from him and put in some kind of an orphanage. Soon he realized he didn't have to pay for our stay if he disowned us. For him, this was easier. All the holidays, all the birthdays … everything was in an orphanage from then on. Other children were going out to be with relatives and such, but not us. This period was difficult for me. I don't like remembering it.

DOMINIC - ANXIOUSNESS

Uh … Tomorrow at half-past one Andrew and I are meeting our mother. I just don't know ... I don't know what to expect, really. Andrew wants this, he found her – I don't even know how you can find a person after twenty years, but he did … And now we have to … have a coffee with her, or something, I guess. What do you even say to a person who just packed up and left? This won't be good, this won't be good at all … or maybe it will be? Maybe it will be great. Maybe she has a good reason and is sorry … But if she was sorry, she would contact us herself, right? She must have known where they put us. I don't know, damn, how do you even prepare for a meeting like this? Andrew is so excited, but I think he will get hurt. And so will I, but I still have to be there, I guess.

ANDREW - HAPPINESS

I think I'm doing much better now. Actually, I think I'm doing well now. Because in high school, I started improving year by year, and now in college, my grades are actually high. And then, I got the scholarship for studying abroad – something I could never even imagine before, because it was completely out of my reach. So, I decided to apply to a few universities that are like, high-quality, prestigious … And this morning, I got this letter. And guess what? I got accepted!

DOMINIC - ANGER

Well, yesterday we met with our mother. It was just as I expected, the woman did not even apologize! And we told her everything! What happened to us after she left, how Brandon beat Andrew so badly he bled from his ear, how they took us and placed us in that rotten orphanage, so we didn't even see the sea until I was fifteen, how we couldn't even have one normal moment in our childhood, all because of her. But no, she sat there completely cool and then had the balls to say that leaving was the best decision of her life! Excuse me? How can you say that to your sons?! And then she even had the nerve to ask Andrew for money when she found out about his scholarship. Is she even sane? I regret falling for this, having to meet the family, yeah. I've had enough!
