# Supplementary material for: Protocol of the study for predicting empathy during VR sessions using sensor data and machine learning
Source: PLoS One. 2024 Jul 18;19(7):e0307385. doi: 10.1371/journal.pone.0307385 (PMC11257359; doi:10.1371/journal.pone.0307385)
Supplement: S3 Appendix — Male version: Jake and Dave. (PDF) [file pone.0307385.s003.pdf]

APPENDIX B

NARRATIVE 2

MALE VERSION: JAKE AND DAVE

JAKE - SADNESS

Actually … okay, let me explain the background first. Dave and I had no contact with our parents for the last three years. We had to cut off contact for our own well-being, really. I know it sounds terrible, but it couldn't continue the way it was. Dad probably has some kind of disorder ... There were constant problems – even if you breathed the wrong way, he would lose his temper. Luckily it was mostly just words, there weren't that many physical fights. But mom was always on his side. We had to cut off contact with her as well, because she didn't want to have a relationship with us without dad. Well … and yesterday, I found out she had a stroke last month. Apparently, she's okay now and on medication. I understand that there are consequences when you cut off contact, but it had to be done … It just isn't fair that we found out just now … and in such a passive-aggressive way from dad … What hurts the most is that mom is blaming us for the stroke and doesn't want to see us. I know it's not my fault that the family isn't functioning, but it really sucks that we can't have normal relationships, and this is happening … The situation just sucks, really. Dave will have a harder time dealing with this.

DAVE - ANXIOUSNESS

This family situation ... I'd say I took it worse than Jake. Well, he had Jane, his girlfriend, to support him all this time, and I had basically no one but him. And I lived at home longer. Because of the problems at home, I always felt different from my classmates. And honestly, I've really messed up my life … It was too much for me, too much was going on, I felt incapable, stupid, everything my dad told me I was. And now I didn't even finish high school, I have no education … I'm working on the production line, and night shifts are really killing me. And yes, I want to change my job, but I don't want to ask Jake for help, because they are expecting a baby and already have so much work on their hands … I don't know what to do; I'm not okay. I have to change something, but I don't know … Every time I think about it, I get so anxious. I don't know …

JAKE - HAPPINESS

Yes, it's true, my girlfriend and I are expecting a baby. She's currently in the eleventh week, and all the checkups have been okay, and Jane is feeling good too. At first, she worried we might have trouble conceiving because of her medical problems, but we did it! And we are both at the point where we have stability and are excited to take this step. Finally, I'm going to have the kind of family I want. This will be something entirely different. When Jane told me … I cried out of happiness. I swear, that has never happened to me before. And she told me she thinks I'll be … a really good dad.

DAVE - ANGER

Jake just told me about mom. You see, this is how my life looks like. I just can't deal with this anymore. Mom had a stroke, and she blames us?! And when we show concern, she shuts us down. Dad completely poisoned her with his sick paranoid ideas! I'm not doing this anymore. Like I hadn't had the whole childhood ruined, like I'm not barely getting by because of them, no, they will make sure to make my life as miserable as they can! Seriously, why would you even decide to have kids if you're going to treat them this way? Is this normal? And in the end, Jake and I are the only ones who feel responsible and guilty for it. No, I've had enough, this has to stop!
