# Supplementary material for: Protocol of the study for predicting empathy during VR sessions using sensor data and machine learning
Source: PLoS One. 2024 Jul 18;19(7):e0307385. doi: 10.1371/journal.pone.0307385 (PMC11257359; doi:10.1371/journal.pone.0307385)
Supplement: S4 Appendix — Female version: Laura and Kate. (PDF) [file pone.0307385.s004.pdf]

APPENDIX B1

NARRATIVE 2

FEMALE VERSION: LAURA AND KATE

<u>LAURA - SADNESS</u>

Actually … okay, let me explain the background first. Kate and I had no contact with our parents for the last three years. We had to cut off contact for our own well-being, really. I know it sounds terrible, but it couldn't continue the way it was. Dad probably has some kind of disorder ... There were constant problems – even if you breathed the wrong way, he would lose his temper. Luckily it was mostly just words, there weren't that many physical fights. But mom was always on his side. We had to cut off contact with her as well, because she didn’t want to have a relationship with us without dad. Well … and yesterday, I found out she had a stroke last month. Apparently, she’s okay now and on medication. I understand that there are consequences when you cut off contact, but it had to be done … It just isn't fair that we found out just now … and in such a passive-aggressive way from dad … What hurts the most is that mom is blaming us for the stroke and doesn’t want to see us. I know it’s not my fault that the family isn’t functioning, but it really sucks that we can’t have normal relationships, and this is happening … The situation just sucks, really. Kate will have a harder time dealing with this.

<u>KATE - ANXIOUSNESS</u>

This family situation ... I’d say I took it worse than Laura. Well, she had Philip, her boyfriend, to support her all this time, and I had basically no one but her. And I lived at home longer. Because of the problems at home, I always felt different from my classmates. And honestly, I’ve really messed up my life … It was too much for me, too much was going on, I felt incapable, stupid, everything my dad told me I was. And now I didn’t even finish high school, I have no education … I’m working on the production line, and night shifts are really killing me. And yes, I want to change my job, but I don’t want to ask Laura for help, because she’s pregnant, and they already have so much work on their hands … I don’t know what to do; I’m not okay. I have to change something, but I don’t know … Every time I think about it, I get so anxious.

<u>LAURA - HAPPINESS</u>

Yes, it’s true, I’m pregnant, did Kate tell you? Now I’m in the eleventh week. All the checkups have been okay, and Philip is really supportive. We’re so happy; we’ve wanted to start a family for a while. I was worried we might have troubles conceiving because of my medical problems, but we did it! And we are both at the point where we have some stability and are thrilled to be taking this step. I’m finally going to create the kind of family I want. This is going to be something entirely different. When I saw the test was positive, I started crying out of happiness. I don’t think this happened to me before. This will finally be a fresh start in my life.

<u>KATE - ANGER</u>

Okay, so Laura just told me about mom. You see, this is how my life looks like. I just can’t deal with this anymore. Mom had a stroke, and she blames us?! And when we show concern, she shuts us down. Dad completely poisoned her with his sick paranoid ideas! I’m not doing this anymore. Like I hadn’t had my whole childhood ruined, like I’m not barely getting by because of them, no, they will make sure to make my life as miserable as they can! Seriously, why would you even decide to have kids if you’re going to treat them this way? Is this normal? And in the end, Laura and I are the only ones who feel responsible and guilty for it. No, I’ve had enough, this has to stop!
