# Supplementary material for: Protocol of the study for predicting empathy during VR sessions using sensor data and machine learning
Source: PLoS One. 2024 Jul 18;19(7):e0307385. doi: 10.1371/journal.pone.0307385 (PMC11257359; doi:10.1371/journal.pone.0307385)
Supplement: S5 Appendix — (PDF) [file pone.0307385.s005.pdf]

APPENDIX C

Personal Information Questionnaire

1. How do you define your gender?
   ☐ Male ☐ Female ☐ Other

2. How old are you?
   ____ years

3. Is your Slovenian fluent?
   ☐ Yes ☐ No

4. Do you suffer from epilepsy or heart condition?
   ☐ Yes ☐ No

5. Do you have any other health conditions (or are using a medicine) that you think could be influencing your heart rate or sweating?
   ☐ Yes ☐ No

6. Would you say your vision is fairly good without glasses (or *with* contact lenses in case you are currently wearing them)?
   ☐ Yes ☐ No

7. Are you aware of having any conditions that are affecting your hearing currently?
   ☐ Yes ☐ No

8. Are you aware of having any conditions that are affecting facial movements such as facial palsy or stroke?
   ☐ Yes ☐ No

9. Are you aware of having any anxiety disorders (e.g., generalised anxiety disorder, panic disorder, social anxiety disorder, PTSD, etc)?
   ☐ Yes ☐ No

10. On a scale from 1-9, please rate your experience with virtual reality?
    (Not at all experienced) (Very experienced)
    1 2 3 4 5 6 7 8 9
    Never tried | Tried a few experiences | 1-2 per month |1-2 times per week | 3+ timer per week

11. What is your current status?
    ☐ Student ☐ Employed ☐ Unemployed ☐ Retired ☐ Other

12. What is the highest degree or level of education you have completed?
    - (un)finished primary school
    - trade school

- high school
- Bachelor's degree
- Master's degree
- Ph.D. or higher

13. Which group your job (if student: study programme) is under?
    - agriculture, food, and natural resources
    - architecture and construction
    - arts, audio/video technology, and communication
    - business, management, administration, and finance
    - education and training
    - government and public administration
    - health science.
    - hospitality and tourism
    - human services
    - information technology
    - law, public safety, corrections, and security
    - manufacturing
    - marketing, sales, and service
    - science, technology, engineering, and mathematics
    - transportation, distribution, and logistics

14. Additional comments about yourself that you think would help the research:
    ____
