# Supplementary material for: Protocol of the study for predicting empathy during VR sessions using sensor data and machine learning
Source: PLoS One. 2024 Jul 18;19(7):e0307385. doi: 10.1371/journal.pone.0307385 (PMC11257359; doi:10.1371/journal.pone.0307385)
Supplement: S6 Appendix — (PDF) [file pone.0307385.s006.pdf]

APPENDIX D

Presence Questionnaire

Participant ID: [……..]

Q1 Presence rating: Did you have a sense of “being there”? (feeling present in the virtual space)

Not at all — Very much

1 2 3 4 5 6 7 8 9

Q2 Did you feel completely captivated by the Virtual Environment?

Not at all — Very much

1 2 3 4 5 6 7 8 9

Q3 How real did the virtual world seem to you?

Not real at all — Completely real

1 2 3 4 5 6 7 8 9

Q4 How aware were you of the real world surrounding while navigating in the virtual world? (i.e. sounds, room temperature, other people, etc.)?

Not aware at all — Extremely aware

1 2 3 4 5 6 7 8 9

Q5 Did you experience any issues that prevented you from experiencing VR properly? (i.e. physical discomfort, poor visual or audio experience due to inability to set the headset properly, nausea, disorientation…)

Not at all — Very much

1 2 3 4 5 6 7 8 9
